# Recalcitrant Pustular Dermatosis Successfully Treated With Prolonged Isotretinoin Therapy

**DOI:** 10.7759/cureus.74501

**Published:** 2024-11-26

**Authors:** Kawaiola Cael Aoki, Will Smithy, Stefanie Au, Simona Bartos

**Affiliations:** 1 Medicine, Dr. Kiran C. Patel College of Osteopathic Medicine, Nova Southeastern University, Fort Lauderdale, USA; 2 Medicine, College of Osteopathic Medicine of the Pacific, Western University of Health Sciences, Pomona, USA; 3 Dermatology, Imperial Dermatology, Hollywood, USA

**Keywords:** dermatoses, isotretinoin, long-term management, pustular dermatosis, pustular rash, systemic retinoids

## Abstract

Isotretinoin (13-cis-retinoic acid) is a well-established systemic treatment for moderate to severe acne vulgaris, renowned for its ability to target multiple contributors to acne pathogenesis. However, its therapeutic potential extends beyond conventional acne management. This case report highlights its efficacy in treating recalcitrant pustular dermatosis, a condition that proved resistant to standard therapies and posed significant diagnostic challenges.

A 25-year-old female patient presented with a diffuse, inflamed pustular rash that was unresponsive to a wide range of conventional treatments, including antibiotics, antifungals, and topical agents. Initially diagnosed as bacterial folliculitis based on clinical appearance, the condition persisted despite these interventions. Multiple punch biopsies and laboratory tests aimed at ruling out other conditions, such as pityriasis folliculorum, eosinophilic folliculitis, and cutaneous mastocytosis, provided inconclusive results. Without a definitive diagnosis and after exhausting standard treatments, the decision to initiate systemic isotretinoin was driven by its unique mechanism of action as a "sebocyte modulator," which targets sebum production, inflammation, and abnormal keratinization-key factors suspected to underlie the patient’s condition.

Over several months, gradual but consistent improvement was observed, culminating in the complete resolution of pustular lesions. The patient’s successful response to isotretinoin highlights the importance of considering this treatment in refractory pustular dermatosis when conventional therapies fail, advocating for its broader clinical application in managing complex pustular skin disorders.

## Introduction

Isotretinoin (13-cis-retinoic acid) is a systemic treatment widely used for moderate to severe acne vulgaris when all conventional methods prove insufficient. As a derivative of retinol or vitamin A, isotretinoin was approved for use by the US Food and Drug Administration (FDA) in 1982. Isotretinoin is the most effective acne treatment to date, with most patients experiencing significant improvements within four to six months of starting treatment. The efficacy and safety of isotretinoin have been extensively studied. Clinical trials and case reports spanning from 1943 to 2019 have demonstrated that 82.6% of patients experienced an improvement in their acne with isotretinoin [1]. Its use has significantly improved the quality of life of many acne patients who have not responded to other treatments. In addition, isotretinoin has a favorable safety profile for non-pregnant patients aged 12 and over, as high doses of vitamin A have been historically used to treat diseases such as Ebola, lung cancer, and COVID-19 [2-4].

Isotretinoin’s mechanism of action targets the four major and recognizable contributors to acne pathogenesis: *Cutibacterium acnes* proliferation, sebum production, pro-inflammatory cytokines, and comedogenesis. Unlike topical treatments, isotretinoin has the unique ability to target the root cause of acne, which is abnormal sebum secretion by sebocytes. Thus, isotretinoin is often referred to as the "sebocyte modulator," as it directly affects the skin's lipid composition. Additionally, isotretinoin has been found to normalize desquamation and reduce inflammation, which also contributes to the development of acne [2].

Diffuse pustular dermatosis presents a unique challenge to dermatologists, demanding a comprehensive understanding of the underlying pathology and its various etiological factors. Conditions like subcorneal pustulosis and pustular psoriasis may present with similar diffuse pustules and inflammation, complicating diagnosis. Pustule formation involves multiple inflammatory signaling cascades, resulting in an accumulation of neutrophils in the stratum corneum [5]. Cytokines, such as interleukin (IL)-36 and IL-8, play a role in the inflammatory process by activating neutrophils and Th17 cells, which further activate neutrophils [6]. This systemic activation of neutrophils in the skin forms a pustule underneath the stratum corneum and can be caused by various systemic diseases and medications, making the diagnosis more difficult. The differential diagnosis of pustular dermatoses includes infectious and autoimmune diseases such as pustular drug reactions, acne vulgaris, rosacea, and folliculitis [7]. Topical benzoyl peroxide has effectively treated pustular drug reactions, while macrolide antibiotics have successfully managed acne vulgaris. Rosacea has responded well to treatments with topical metronidazole and sodium acetazolamide. Folliculitis treatments include topical antibiotics such as mupirocin and ceftriaxone, chosen based on the specific bacterial strain involved [7]. Hence, differentiating between localized and diffuse pustular rashes and considering the age and gender of the patient help narrow down the potential diagnoses. By carefully considering these factors, clinicians can successfully develop effective treatment strategies to manage this condition.

This case study demonstrates the potential therapeutic utility of isotretinoin beyond its conventional indication for acne vulgaris. By shifting from a theoretical framework to a clinical one, we illustrate the pharmacologic effect of isotretinoin in treating recalcitrant pustular rashes, particularly those resistant to common treatments such as antibiotics and antifungals. This study highlights the versatility of isotretinoin as a promising treatment option for pustular dermatological conditions, particularly in cases where other therapies have failed.

## Case presentation

**Figure 1:**
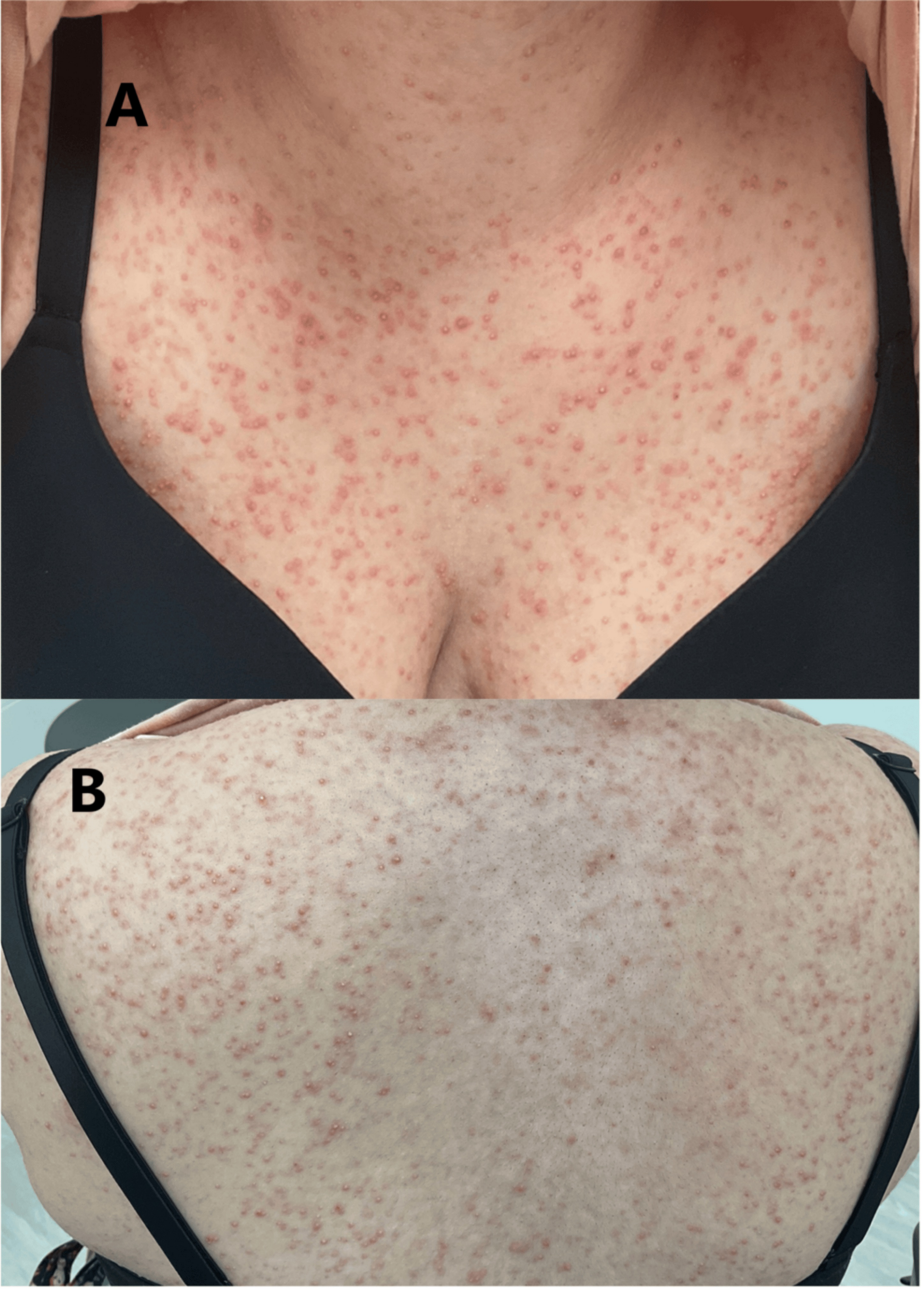
(A,B) Initial presentation of diffuse pustular rash on chest and back

A 25-year-old female patient presented to the clinic with a diffuse, moderately inflamed, and pruritic rash that had persisted for several months, with no systemic complaints or comorbidities. On physical examination, folliculocentric pink pustules and papules with minimal scaling were observed on her head, neck, torso, pelvis, and extremities (Figure 1). She was diagnosed with folliculitis and prescribed a comprehensive treatment regimen targeting potential fungal, bacterial, and inflammatory causes. Ketoconazole 2% shampoo and cream, along with fluconazole 200 mg daily for two weeks, followed by 150 mg weekly, and miconazole 2% topical cream were given to manage possible fungal involvement. Doxycycline 100 mg twice daily was prescribed for its antibacterial and anti-inflammatory properties. To further address inflammation and alleviate pruritus, clobetasol 0.05% topical solution and triamcinolone 0.1% cream were prescribed, and fexofenadine 180 mg four times daily was recommended to reduce itching. Additionally, chlorhexidine gluconate 4% topical was included to lower skin bacteria levels and prevent infection.

**Figure 2:**
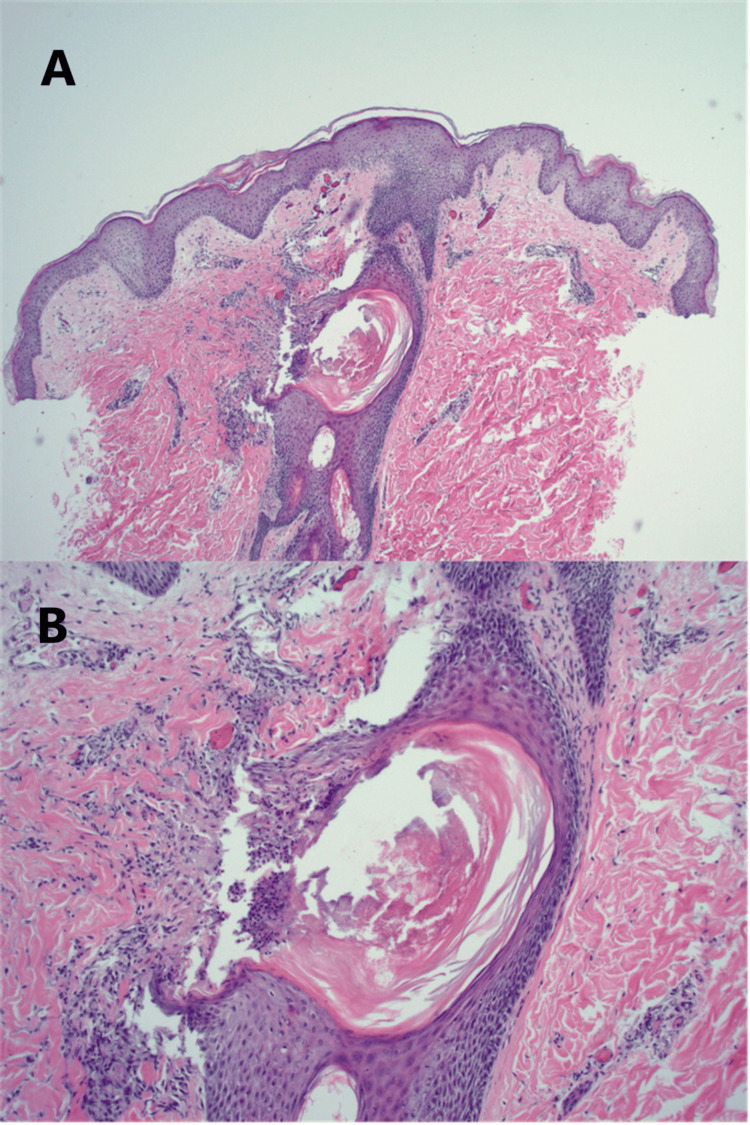
(A,B) Punch biopsy from the left lateral chest infra-axillary area showing acneiform folliculitis (H&E, 4x, 10x)

One month later, she reported no improvement in her rash. Two punch biopsies were obtained to rule out pityriasis folliculorum and eosinophilic folliculitis. Mild chronic perifolliculitis with pityrosporum-like organisms and acneiform folliculitis were identified (Figure 2). Neutrophil cytoplasmic antigen (NCA) staining did not demonstrate dermal mast cell proliferation consistent with cutaneous mastocytosis. No pathogenic bacteria grew on cultures. The patient was prescribed trimethoprim-sulfamethoxazole (Bactrim DS) 800 mg-160 mg twice daily for 14 days and ketoconazole 2% cream twice daily.

**Figure 3:**
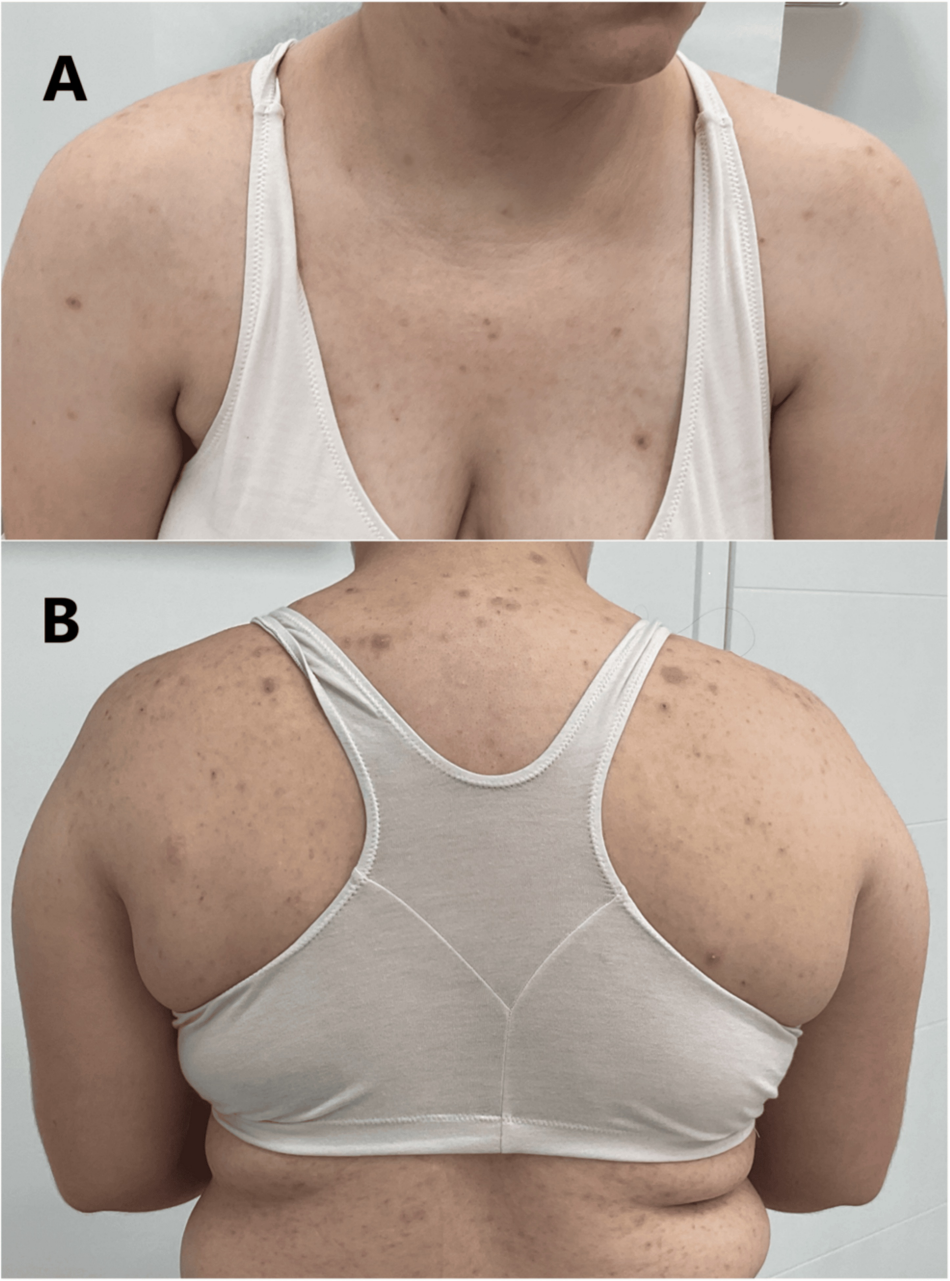
(A,B) Recalcitrant inflamed erythematous papules and pustules four months after initial presentation

The patient's rash continued to be non-responsive to treatment. A myriad of open and closed comedones, inflamed pink erythematous papules, pustules, nodules, cysts, and scarring on the chest, face, and upper arms continued to be seen (Figure 3). Labs were obtained, and the patient was counseled on systemic isotretinoin. She was started on 20 mg isotretinoin twice daily.

**Figure 4:**
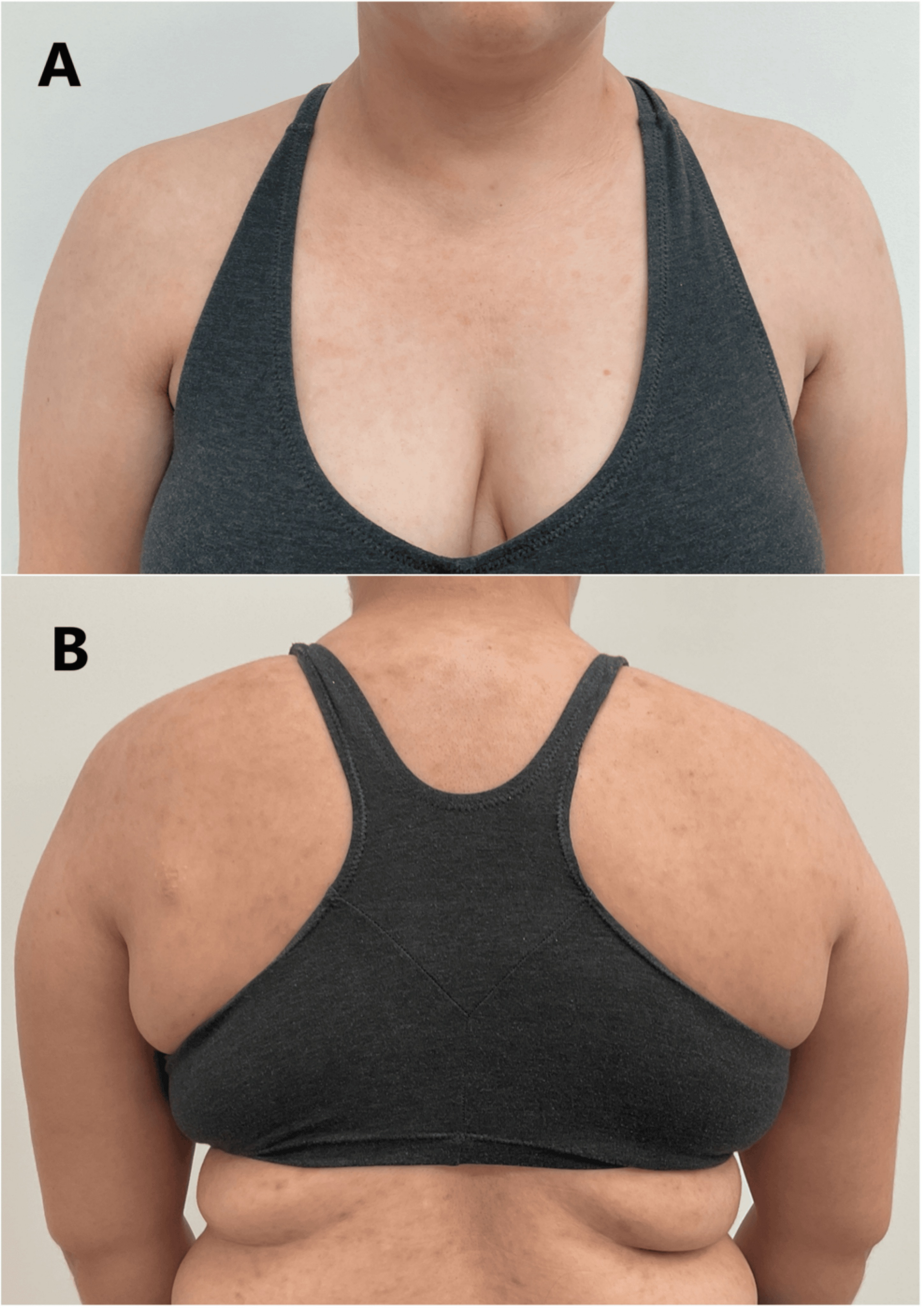
(A,B) Improvement in pustular rash after seven months of isotretinoin

For month two, the dosage was increased to 30 mg twice daily, and she was maintained at that dose through month seven. Improvement in her condition was noted at this time (Figure 4). The dose was decreased to 20 mg twice daily for month eight. The patient reported continual improvement, and on month nine, her treatment regimen was supplemented with clascoterone 1% (Winlevi) cream and clindamycin-benzoyl peroxide 1.2%-5% topical gel.

**Figure 5:**
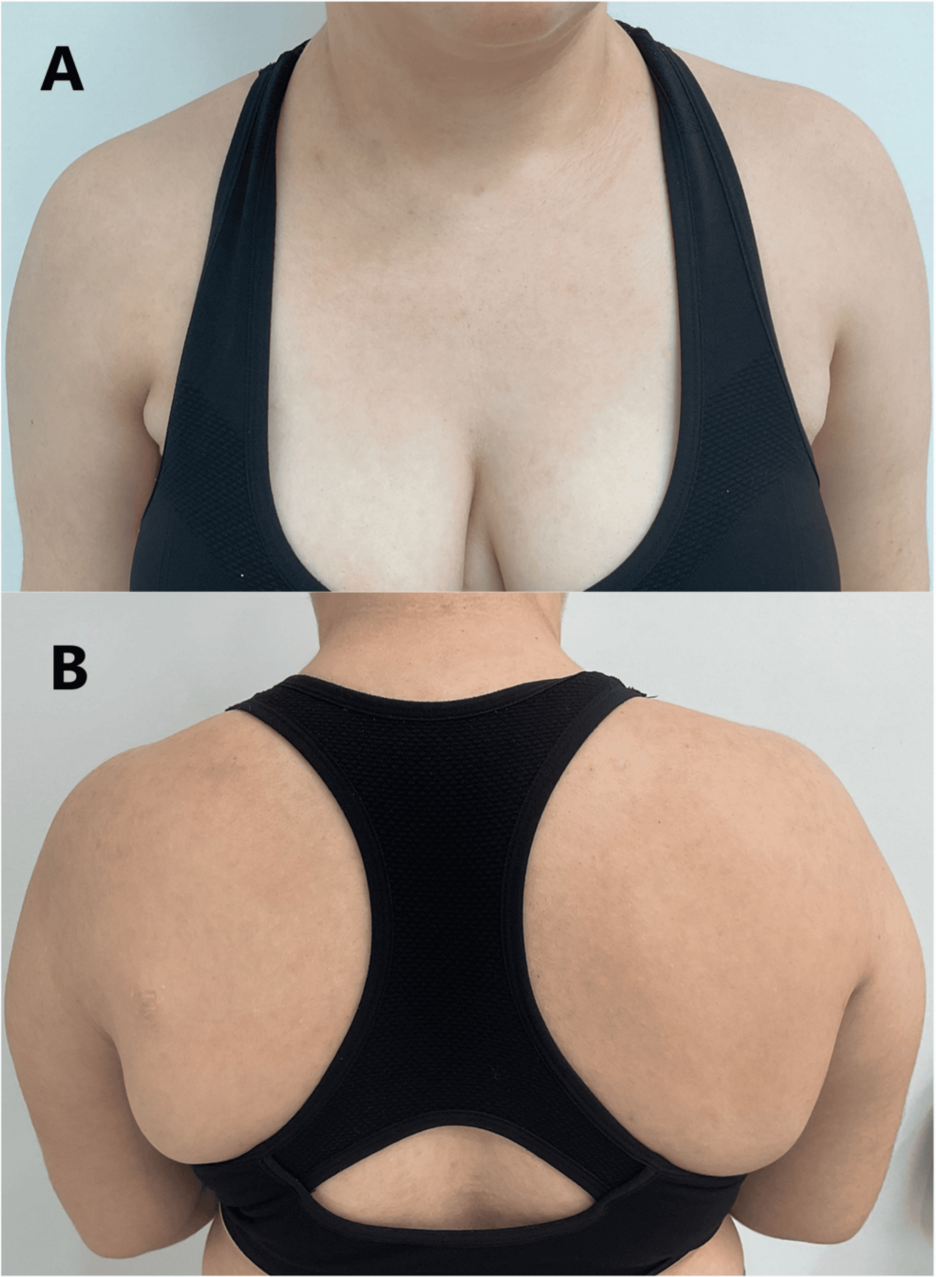
(A,B) Complete resolution of pustular dermatosis after 20 months of isotretinoin

From months 10 to 19, her dose of isotretinoin was reduced to 10 mg daily and 10 mg every other day from months 19 to 20. On month 19, no subcutaneous nodules, cysts, or pink erythematous papules/pustules were seen (Figure 5). The patient completed 20 months of isotretinoin treatment, which included a therapeutic period followed by a very slow taper. Follow-up showed no recurrence of pustular lesions, suggesting durability in the response to isotretinoin.

## Discussion

The overall approach to a diffuse pustular rash can vary, as can the different treatment options. Clinically, diffuse pustular rashes may not give a definitive result on histology or by just their clinical presentation. However, as the current case illustrates, recalcitrant folliculitis and other pustular rashes may respond favorably to isotretinoin, offering a viable alternative when other therapies fail.

Three main types of pustular rashes are typically generalized or diffuse-pustular psoriasis, Reiter's disease, and subcorneal pustular dermatosis, or Sneddon-Wilkinson disease. Additionally, there are also medication-induced generalized pustular rashes, such as acute generalized exanthematous pustulosis and acneiform drug eruptions [7]. Generalized pustular dermatosis can be fatal and is usually characterized by a rapid onset of pustules accompanied by fever, arthralgias, and myalgias. Patients over the age of 40 with a history of psoriasis are typically affected, which contrasts with our patient, who was 25 years old and had no history of psoriasis. There are a variety of triggering factors that can lead to this type of rash. A combination of immunosuppressants and specific biologics targeting psoriasis are used to manage the symptoms [7]. Despite the severity of her symptoms, our patient lacked systemic symptoms, such as fever and joint pain, typically associated with generalized pustular psoriasis, and thus, this diagnosis was ruled out. However, there have been successful cases of treating this condition with isotretinoin, including in a child [8].

Reiter's disease is an autoimmune condition typically associated with a prior gastrointestinal or urinary tract infection. This condition is characterized by a combination of symptoms such as conjunctivitis and enthesopathy, which is inflammation of the tendons and ligaments. Given the absence of recent infections, conjunctivitis, or musculoskeletal symptoms, Reiter’s disease was considered unlikely.

On the other hand, subcorneal pustular dermatosis is a chronic pustular disorder that is commonly diagnosed in middle-aged women. This condition is usually confirmed by subcorneal collections of polymorphonuclear leukocytes on histology, and it can be effectively treated with the medication dapsone [7]. In this case, our patient’s age and biopsy findings did not match the typical profile for subcorneal pustular dermatosis, further ruling out this condition.

While treatments like biologics and immunosuppressants are often used to manage cases of diffuse pustular dermatoses, isotretinoin’s unique mechanism of targeting sebum production, inflammation, and keratinization offers a distinct approach with specific advantages in refractory cases. Retinoids, including isotretinoin, inhibit the differentiation of Th17 cells while promoting T regulatory cell expression, thereby fostering an anti-inflammatory response. In addition, retinoids may reduce the production of key inflammatory cytokines, such as TNFα, IL-1, and IL-6, which are implicated in the pathology of pustular dermatoses [9]. This immune-modulating effect likely contributed to the therapeutic success observed in this case by mitigating the inflammatory environment supporting pustule formation.

By contrast, biologics often require regular administration by injection and may carry risks of infection or systemic side effects due to their modulation of immune pathways. While biologics target specific immune pathways, such as TNFα in TNF inhibitors, isotretinoin’s broader impact across multiple inflammatory pathways may provide a more comprehensive approach in certain refractory cases. In this case, the decision to use isotretinoin highlights the distinct approach of targeting multiple inflammatory pathways-unlike biologics, which typically focus on specific cytokines-thereby addressing the broader inflammatory mechanisms that contribute to sustained resolution in complex pustular conditions.

In the face of diagnostic challenges surrounding the presented rash, our clinical approach prioritized critical thinking and a nuanced understanding of the underlying pathology. Despite initial difficulties in pinpointing a definitive diagnosis, the persistence of the rash, unresponsive to conventional therapies, prompted a strategic shift toward addressing the observed pustules specifically. Recognizing isotretinoin's unique mechanism of action as a “sebocyte modulator,” capable of intervening in abnormal sebum secretion, pro-inflammatory cytokines, and comedogenesis, guided this decision. This therapeutic pivot aligns with the notion that treating the root cause, rather than relying solely on symptomatic relief, can yield transformative outcomes. The subsequent success observed in the patient's response to isotretinoin reinforces the significance of tailored treatment strategies informed by a comprehensive understanding of the pathogenesis to guide therapeutic decisions when faced with diagnostically elusive dermatological conditions.

## Conclusions

The presented case study demonstrates the efficacy of isotretinoin in treating recalcitrant pustular dermatosis. This case contributes to the broader discourse on the diverse etiologies of pustular rashes and the nuanced treatment strategies required for resolution. Isotretinoin, known for its unparalleled impact on acne, demonstrated its potential as an effective therapeutic agent in addressing this complex dermatological condition. The successful outcome observed in this case suggests that isotretinoin, with its unique mechanism of action, holds promise in managing challenging pustular skin disorders. The durability of the patient’s response post-treatment supports isotretinoin's use in similar refractory cases. This case also highlights a need for further research, including clinical trials, to explore isotretinoin’s application in pustular dermatoses and identify potential biomarkers that could predict a positive response to isotretinoin.

## References

[1] Cook MK, Perche PO, Feldman SR (2022). The use of oral vitamin A in acne management: a review. Dermatol Online J.

[2] Webster GF (2015). Isotretinoin: mechanism of action and patient selection. Semin Cutan Med Surg.

[3] Aluisio AR, Perera SM, Yam D (2019). Vitamin A supplementation was associated with reduced mortality in patients with Ebola virus disease during the West African outbreak. J Nutr.

[4] Somi MH, Faghih Dinevari M, Taghizadieh A, Varshochi M, Sadeghi Majd E, Abbasian S, Nikniaz Z (2024). Effect of vitamin A supplementation on the outcome severity of COVID-19 in hospitalized patients: a pilot randomized clinical trial. Nutr Health.

[5] Wang H, Jin H (2021). Update on the aetiology and mechanisms of generalized pustular psoriasis. Eur J Dermatol.

[6] Sugiura K (2022). Role of interleukin 36 in generalised pustular psoriasis and beyond. Dermatol Ther (Heidelb).

[7] Mengesha YM, Bennett ML (2002). Pustular skin disorders: diagnosis and treatment. Am J Clin Dermatol.

[8] Al-Shobaili H, Al-Khenaizan S (2007). Childhood generalized pustular psoriasis: successful treatment with isotretinoin. Pediatr Dermatol.

[9] Kodali N, Blanchard I, Kunamneni S, Lebwohl MG (2023). Current management of generalized pustular psoriasis. Exp Dermatol.

